# Improving protein hydrolysis and digestibility in *Arthrospira platensis* biomass through recombinant peptidases (EC 3.4): Opportunities for monogastric animal diets

**DOI:** 10.1016/j.heliyon.2024.e41460

**Published:** 2024-12-25

**Authors:** Maria P. Spínola, Mónica M. Costa, Rita S. Simões, Vânia O. Fernandes, Vânia Cardoso, Virgínia M.R. Pires, Cláudia Afonso, Carlos Cardoso, Narcisa M. Bandarra, Carlos M.G.A. Fontes, José A.M. Prates

**Affiliations:** a CIISA - Centro de Investigação Interdisciplinar em Sanidade Animal, Faculdade de Medicina Veterinária, Universidade de Lisboa, Av. da Universidade Técnica, 1300-477, Lisboa, Portugal; b Associate Laboratory for Animal and Veterinary Sciences (AL4AnimalS), Faculdade de Medicina Veterinária, Universidade de Lisboa, Av. Da Universidade Técnica, 1300-477, Lisboa, Portugal; c NZYTech - Genes and Enzymes, Estrada do Paço do Lumiar, Campus do Lumiar, Edifício E, 1649-038, Lisboa, Portugal; d DivAV - Division of Aquaculture and Upgrading, Portuguese Institute for the Sea and Atmosphere, Rua Alfredo Magalhães Ramalho, 6, 1495-006, Lisbon, Portugal; e CIIMAR, Interdisciplinary Centre of Marine and Environmental Research, University of Porto, Rua dos Bragas 289, 4050-123, Porto, Portugal

**Keywords:** *Arthrospira platensis*, Microalga, Protein hydrolysis, Protein digestibility, Recombinant peptidase

## Abstract

This study investigates the use of recombinant peptidases (EC 3.4) to improve protein hydrolysis and digestibility in *Arthrospira platensis*, with a focus on addressing the challenge of reduced protein bioavailability for monogastric animals due to resistant protein-pigment formations, such as phycocyanin, and increased digesta viscosity caused by jellification properties. A library of 192 peptidases was generated, from which 142 soluble peptidases were expressed in *Escherichia coli* and subsequently screened for activity against an *A. platensis* suspension *in vitro*. Among these peptidases, six promising candidates were identified for protein and peptide extraction from the microalga. These enzymes were tested individually, and in a mix (MIX6), and compared to commercial trypsin and pancreatin. Protein content was determined using the Bradford method and potential peptide formation was measured via an *o*-phthaldialdehyde (OPA) assay. The protein solubility and hydrolysis, specifically of two main protein fractions (18–26 kDa and 40–48 kDa) along with minor fractions, were analysed via 14 % sodium dodecyl sulphate-polyacrylamide gel electrophoresis (SDS-PAGE). Results indicated that the enzyme ID 138, a serine-peptidase, significantly increased total peptide formation in the *A. platensis* supernatant, although it did not outperform other peptidases or enzyme mixtures. Notably, enzymes ID 152, derived from a marine bacterium, and ID 153, another serine-peptidase, exhibited significant improvements in the extraction and hydrolysis of one protein fraction (18–26 kDa), possibly corresponding to a phycocyanin fraction. While no synergistic effects were observed among peptidases, further investigations are warranted to understand the enzyme composition of MIX6, particularly enzymes ID 138, ID 152 and ID 153, and their potential to enhance the bioavailability of *A. platensis* proteins for monogastric animals when incorporated into dietary formulations.

## Highlights

• Six promising candidates were identified for protein and peptide extraction from the microalga.
• Enzyme ID 138, a serine-peptidase, significantly increased total peptide formation in the *A. platensis* supernatant.
• Enzymes ID 152 and ID 153 improvemed in the extraction and hydrolysis of protein fraction 18–26 kDa.

## 1 Introduction

Microalgae, particularly *Arthrospira platensis*, an exemplary blue-green algae (*Cyanophyceae*), currently have attracted considerable interest due to their versatile applications for feed, food and other industry purposes [1]. Notably, *A. platensis* stands out with its remarkable protein content, constituting up to 76 % of its dry matter, and richness in bioactive compounds, including pigments [1]. However, the potential bioaccessibility and bioavailability of these highly nutritious compounds are significantly restricted by the microalga's robust cell wall [1].

The cell wall of *A. platensis* predominantly comprises insoluble carbohydrates, specifically glucan and peptidoglycan polymers, which are arrayed in a multi-layered structure cloaked by acidic polysaccharides [2]. A proposed structural model suggests amino acid residues bridging the carbohydrate chains of peptidoglycans, forming peptide bonds within *A. platensis'* cross-walls [3]. Moreover, the cell wall contains neutral monosaccharides and uronic acid residues as polysaccharide constituents, with some structured into extracellular polymeric substances on the outer layer, contributing to increased medium viscosity [4].

Notably, the primary algal proteins form resilient complexes with pigments, known as phycocyanins, belonging to the light-harvesting phycobiliprotein family. These complexes are composed of trimeric or hexameric assemblies of *c*-phycocyanin and trimeric structures of allophycocyanin [5,6]. The binding of several phycobiliproteins constructs phycobilisomes, which are anchored to the thylakoid membrane of microalgae [5,6]. These characteristics present substantial obstacles to the digestion and nutrient absorption of *A. platensis* in monogastric animals, highlighting the need to fully unlock its nutritional potential [1].

Exploration into mechanical/physical pre-treatments, including ultrasonication, with or without enzymatic intervention, has yielded promising results in degrading the cell wall of *A. platensis* and subsequently extracting algal proteins [1]. Safi, Ursu [7] reported a commendable protein extraction yield of 47.1 % using ultrasonication. Incorporating ultrasonication with other mechanical/physical procedures such as agitation [8] or freeze-thaw cycles [9], has been shown to further enhance the extraction of total protein and phycocyanin, yielding up to 75.8 % [8] and 93.1 % [9], respectively. The utilization of ultrasonication in combination with lysozyme also demonstrated effective extraction of allophycocyanin (44.1 g/kg biomass; 80 % yield) [10].

The exclusive application of enzymatic treatments on *A. platensis* biomass has showcased their efficacy in extracting algal proteins. An *in vitro* study [11] documented a 1.34‐fold increase of soluble protein from *A. platensis* post- Carbohydrate-Active Enzyme mixture treatment with recombinant lysozyme and α-amylase, which partially disrupted the cell wall. Similarly, an *in vitro* digestion of *A. platensis* with pepsin and pancreatin led to 81 % digestibility of crude protein [12]. Nevertheless, high protein content release from microalgal biomass might instigate protein jellification due to its gel-forming properties [13,14], potentially impeding nutrient digestion and absorption by inducing increased digesta viscosity in monogastric animals fed a high level of *A. platensis* (>10 % feed), particularly broiler chickens [13,14].

While the hydrolysis of *A. platensis* proteins remains a challenge, past research efforts have endeavoured to degrade these proteins, predominantly for bioactive peptide production. Aiello, Li [15] detected *α*- (18.1 kDa) and *β*-subunits (17.6 kDa) of *c*-phycocyanin (20 % of dried biomass) in ultrasound-treated *A. platensis* extract, which were subsequently hydrolysed into peptides using trypsin or pepsin. In parallel, Böcker, Hostettler [5] noted primary protein fragments ranging from 17 to 20 kDa (*c*-phycocyanin and allophycocyanin subunits) in a purified extract of *A. platensis*, vulnerable to denaturation at elevated temperatures (50–70 °C).

Recently, studies by Otero and Verdasco-Martín [16] and Villaró, Jiménez-Márquez [17] described the hydrolytic activity of several enzymes, including Alcalase® (a subtilisin Ca^2^⁺-dependent serine endoprotease) [18], papain (a sulfhydryl protease from the C1A peptidase family) [19], ficin (a proteolytic enzyme from the latex of *Ficus* species belonging to the C1A family) [20], and pepsin (a member of the A1 peptidase family, EC 3.4.23.1). These enzymes have demonstrated effectiveness in hydrolysing *A. platensis* proteins, including phycobiliproteins such as phycocyanin. Alcalase® is derived from microbial sources, papain and ficin are plant-derived and pepsin originates from animal sources [21]. Collectively, these enzymes are widely used to hydrolyse proteins from meat muscle and by-products, underscoring their utility in biotechnological applications.

This study aimed to generate and evaluate a comprehensive set of 192 recombinant peptidases, assessing their individual and combined effectiveness in the extraction and hydrolysis of *A. platensis* proteins post-mild sonication pre-treatment. By breaking down the resistant cell wall and protein-pigment complexes, this research seeks to unlock the valuable nutrients in *A. platensis* for application in monogastric animal diets.

## 2 Material and methods

### 2.1 Selection, synthesis and cloning of recombinant enzymes’ genes and their expression

A comprehensive library comprising 192 peptidases was established using an extensive collection listed in MEROPS. This collection included all proteolytic enzyme families, namely aspartic-, cysteine-, metal- and serine-type peptidases, each capable of hydrolysing *A. platensis* proteins. The ensuing steps of creating recombinant plasmids, along with expressing and purifying the encoded peptidases, followed the methodology proposed by Coelho, Lopes [22].

The genes coding for the selected peptidases were synthesized using the NZYGene Synthesis kit (Nzytech, Lisbon, Portugal). The sequences of these recombinant peptidases and their encoding DNA are detailed in Supplementary Table 1. Direct cloning of these synthetic genes was accomplished using the NZYEasy Cloning & ExpressKit I kit (Nzytech, Lisbon, Portugal) and the pHTP 1 vector (Nzytech, Lisbon, Portugal), after optimizing the genes for cloning and expression in *Escherichia coli*. Following this, the recombinant plasmids were transformed into *E. coli* BL21 (DE3) cells in microplates. As many as ninety-six peptidases were then concurrently purified using Immobilised Metal Affinity Chromatography (IMAC) by an automated protocol [23], albeit with specific modifications as previously reported [24,25].

To confirm the expression, molecular mass, and solubility of the recombinant enzymes, sodium dodecyl sulphate-polyacrylamide gel electrophoresis (SDS-PAGE) was employed on 14 % (w/v) acrylamide gels. A low molecular weight (LMW) protein marker (18.5–96 kDa) (Nzytech, Lisbon, Portugal) was utilized for the comparison of molecular mass. Gel images were subsequently captured through a dedicated imaging system (BioRad ChemiDoc XRS; Bio-Rad, Hercules, CA, USA). The concentration of enzyme stock solutions, crucial for assessing protein expression level, was determined by spectrophotometry using NanoDrop 2000c (Thermo Fisher Scientific, Pittsburgh, PA, USA) [24,25], reaching a maximum value of 3.9 g/L.

### 2.2 Preparation of A. platensis biomass suspension and *in vitro* enzymatic screening

The spray-dried powder of *A. platensis*, sourced from Allmicroalgae Natural Products SA Company (Pataias, Leiria, Portugal), was securely stored at −20 °C until required for use. Upon use, the microalga was suspended in a phosphate-buffered saline solution (1 × PBS, BioWhittaker, Verviers, Belgium) at a concentration of 20 g/L and subjected to a sonication procedure as recently described [26].

For assessing the hydrolytic capacity of the peptidases on microalga protein, a preliminary *in vitro* enzymatic screening was conducted twice, followed by a final trial in triplicate with enzyme mixtures. The biomass of *A. platensis* was incubated in a microplate along with the enzymes at a concentration of 20 mg/L [24,25]. The specific peptidases utilized are detailed in Supplementary Table 1.

In addition to the recombinant peptidases, commercially available trypsin and pancreatin powders derived from the porcine pancreas were examined for their potential to degrade algal proteins. Trypsin (type II-S, 1000–2000 units/mg dry weight, Sigma-Aldrich, St Louis, MO, USA) was trialled and contrasted with the recombinant peptidases across all assays. Concurrently, pancreatin (350 FIP-U/g peptidase, 6000 FIP-U/g lipase, 7500 FIP-U/g amylase; Merck, Darmstadt, Germany) was incorporated in the final assay.

Upon completion of these steps, each microplate was centrifuged for 15 min at 3210 g, and 1 mL of the supernatant, considerer the hydrolysate, from each replicate was meticulously collected for subsequent analysis.

### 2.3 Determination of total protein released from A. platensis biomass

The total protein present in the algal supernatant was evaluated by spectrophotometry utilizing the Bradford method [27], with adjustments as detailed in recent research [26,28]. In brief, 30 μL of the sample, diluted to a ratio of 1:3 in a PBS solution, was mixed with 1.5 mL of Bradford solution. Following a 5-min resting period at room temperature, absorbance was measured at 595 nm. A standard curve was also prepared and used for this assessment.

The calculation of total soluble protein extraction yield was adjusted from the methodology proposed by Postma, Miron [29] and is outlined as follows:

Proteinextractionyield(%)=100×[(CpTRAT/CpMI)−(CpCON/CpMI)]

In this equation, CpTRAT corresponds to the protein concentration (g/L) achieved with enzymatic treatments; CpCON represents the protein concentration (g/L) achieved with the control; and CpMI refers to the protein concentration (g/L) in the microalga biomass.

The protein concentration in the microalga biomass was determined based on a crude protein value of 58.9 % dry weight, a value obtained from the *A. platensis* biomass and provided by the Allmicroalgae Company. This was calculated for a microalga suspension concentration of 20 g/L.

### 2.4 Assessment of total peptides produced from A. platensis proteins

The total peptides and free amino acids were evaluated using spectrophotometry based on an *o*-phthaldialdehyde (OPA) assay. This procedure, first detailed by Sedighi, Jalili [30] and Vizcaíno, Sáez [31], was slightly modified as documented recently by Costa, Spínola [26] and Spínola, Costa [28]. In summary, the protein present in the algal supernatant was precipitated with 20 % trichloroacetic acid. Following precipitation, the sample was centrifuged, and 200 μL of the supernatant was collected. This supernatant was then combined with 1 mL of the OPA reagent for further analysis.

### 2.5 Determination of solubility and hydrolysis of A. platensis protein fractions

The solubility and hydrolysis of *A. platensis* protein fractions were analysed using SDS-PAGE, a technique recently implemented for similar assessments [26,28]. For this analysis, supernatant samples were loaded in triplicate onto 14 % polyacrylamide gels. A low molecular weight (LMW) protein marker, added at a volume of 5 μL, provided a reference for molecular mass comparison. Subsequently, the resulting gel images were captured using ChemiDoc XRS + software (Bio-Rad Laboratories, Inc., CA, USA). The relative densities of protein fractions within the 18–26 kDa and 40–48 kDa ranges, as well as other minor protein fractions, were quantified with the Image J software (version 1.53s; NIH, Bethesda, MA, USA).

The selection of these fractions was based on their prevalence in the gel and their relationship with specific proteins previously reported in *A. platensis*. Specifically, the 18–26 kDa fraction likely contained the α- and β-subunits of allophycocyanin and *c*-phycocyanin [5,15]. Meanwhile, the 40–48 kDa fraction corresponded to yet unidentified proteins, although they predominated among the higher molecular weight proteins in the sample.

### 2.6 Coefficients of protein degradation analysis for A. platensis

The Coefficients of Protein Degradation (CPD) values were ascertained for protein fractions of *A. platensis*, following a methodology previously proposed [31,32] and adapted by Costa, Spínola [26]. This analysis offers an assessment of the extent of algal protein degradation by comparing protein concentrations before and after enzymatic hydrolysis.

The pixel intensity (in arbitrary units) of the total protein, the two major protein fraction bands, and other minor protein fractions was measured using Image J software (version 1.53s; NIH, Bethesda, MA, USA) in SDS-PAGE gels stained with Coomassie Blue. This data was then compared to the intensity of the bands from a low molecular weight (LMW) protein marker, which corresponded to a total protein concentration of 1.8 g/L, as provided by the manufacturer. The results were ultimately reported in percentages, allowing for a clear, quantitative assessment of protein degradation.

### 2.7 Data statistical analysis

Data analysis was conducted using the Generalised Linear Mixed (GLM) model in the SAS software package (version 9.4; SAS Institute Inc., Cary, NC, USA). To address multiple comparisons, both ANOVA and the Tukey-Kramer method (PDIFF option) were employed, adjusting least-square means in the process. For the assessment of normality and homogeneity of variance, the Shapiro-Wilk and Levene's tests were respectively utilized. Results are presented as mean values accompanied by the standard error of the mean (SEM). A *P*-value of less than 0.05 was considered statistically significant.

## 3 Results

### 3.1 Selection and evaluation of expression and purity of recombinant peptidases

A comprehensive library comprising 192 peptidases (EC 3.4), representative of all proteolytic enzyme families, carefully chosen based on homology covering aspartic-, cysteine-, metal- and serine-type peptidases. MEROPS accession numbers for these peptidases are presented in Supplementary Table 1. Overall, 60 enzymes were sourced from marine or freshwater organisms, and 72 were thermophile-derived (24 from marine hydrothermal origin). The other 60 peptidases were produced by non-marine and non-thermophilic organisms. While the selection predominantly favoured endopeptidases, 9 oligo- or exopeptidases were included due to their marine (e.g., *Aquimarina agarilytica* or *Zobellia* sp.) or thermophilic (e.g., *Sulfolobus solfataricus* or *Thermococcus litoralis*) bacterial origins and homology with other peptidases. Moreover, the characterization of 47 enzymes remains incomplete, with 4 belonging to uncharacterized families, 13 being predictable metallopeptidase-like enzymes, and 30 targeting unknown substrates.

Nineteen peptidases were chosen for their predicted activity on peptidoglycan/murein (alternating strands of *β*-1, 4-linked N-acetylglucosamine and N-acetylmuramic acid) or other long-chain polymers containing N-acetylglucosamine such as chitin, which was recently reported in the *Chlorella vulgaris* cell wall by Weber, Grande [33]. Additionally, 8 peptidases homologous to trypsin or chymotrypsin were selected due to their broad spectrum of activity, compared to other enzymes with specific action, as spr peptidase (peptidoglycan).

The primary sequences of these peptidases were designed, synthesized, and cloned into the prokaryotic expression vector pHTP1. To optimize their expression in *E. coli*, codon usage for the recombinant genes was adjusted accordingly. Utilizing high- and medium-throughput protocols, the expression and purification of recombinant peptidases were facilitated. The expression level of soluble enzymes was evaluated using a qualitative scale based on protein concentrations (g/L): , 0.0 > 0.1; +, 0.1 ≥ 0.6; ++, 0.6 ≥ 1.6; +++, 1.6 ≥ 2.6; ++++, >2.6 (Supplementary Table 1).

Of the 192 recombinant enzymes, 31 did not express, and 19 were insoluble, thereby excluded from initial screening. Consequently, 142 peptidases and commercial trypsin were tested for their protein hydrolysis ability in *A. platensis*. Before the initial screening of recombinant enzymes, a high-throughput IMAC protocol was used to enrich soluble protein fractions. The expression varied among these enzymes: 106 showed low expression (+), 44 moderate expression (++), and 11 high expression (+++, ++++). However, one high-expression enzyme had low solubility. Nineteen peptidases exhibited unexpected migration patterns in SDS-PAGE based on their molecular weights (MW), with some having lower MW and others having higher MW than anticipated. The lysis of the *E. coli* cell wall, attributable to peptidase activity in 26 % of cases, likely caused the non-expression (concentration below 0.1 g/L) of proteins quantified after purification (Supplementary Table 1 and Supplementary Figs. 1A and B).

### 3.2 Initial screening of recombinant peptidases for A. platensis’ protein hydrolysis

The incubation of each recombinant enzyme with the *A. platensis* suspension revealed that most peptidases were unable to hydrolyse microalgal proteins or release proteins and peptides into the algal supernatant (Supplementary Table 2). However, 6 enzymes (ID 113, 121, 138, 152, 153 and 159), in addition to the commercial trypsin, successfully extracted total protein or peptides from *A. platensis* biomass. These were measured using the Bradford method and OPA spectrophotometric assay, respectively.

Then, data were organised according to two qualitative scales (Table 1): 1) increased total protein concentration (g/L): , ≤0.05; +, 0.05 ≥ 0.10; ++, 0.10 ≥ 0.15; +++, 0.15 ≥ 0.20, ++++, >0.20; and 2) increased peptide concentration (mg/L): , ≤1.0; +, 1.0 ≥ 4.0; ++, 4.0 ≥ 6.0; +++, 6.0 ≥ 8.0; ++++, >8.0.

**Table 1:** Screening of the selected individual peptidases for the extraction and hydrolysis of *Arthrospira platensis* proteins.

ID^a^	Name	Category	EC number	Main substrate	Increased protein concentration^b^	Increased peptide concentration^c^
113	ShyA D,D-endopeptidase (*Vibrio cholerae* type)	Metallopeptidase	EC 3.4.24.-	Peptide chain cross-links in *Vibrio cholerae* ‘s peptidoglycan	+ (0.08)	++++ (9.04)
121	ybbE (*Bacillus subtilis* type)	N-acetylmuramoyl-L-alanine amidase	EC 3.5.1.28	Hydrolyses link between N-acetylmuramoyl and L-amino acid residues in certain cell-wall glycopeptides	+ (0.06)	+++ (7.89)
138	Htra2 peptidase (*Mycobacterium* type)	Serine-type peptidases	EC 3.4.21.-	Substrate with cleavage site at peptide-Ala148+Ala-peptide; peptidase S1 and S6 chymotrypsin/Hap	++ (0.13)	++++ (8.09)
152	Hypothetical protein PULV_a1212	Uncharacterized peptidase	EC 3.4.-. -	Unknown substrate of bacterium isolated from *Ulva lactuca* surface	+++ (0.19)	-(-5.55)
153	MCP-01 peptidase	Serine-type peptidases	EC 3.4.21.-	Unknown; subtilisin-like peptidase	+++ (0.16)	-(-0.26)
159	PF1438 (*Pyrococcus furiosus* holotype)	Serine-type peptidases	EC 3.4.21.-	Unknown; predicted archaeal peptidase of S18 family with subunit ChlI of Mg-chelatase	++++ (0.21)	-(-2.49)
Commercial^d^	Trypsin	Serine-type peptidase	EC 3.4.21.4	Preferential cleavage of Arg-|-Xaa, Lys-|-Xaa	-(-0.11)	++++ (10.6)

a Project identification number (ID).

b Increased total protein concentration (g/L): , ≤0.05; +, 0.05 ≥ 0.10; ++, 0.10 ≥ 0.15; +++, 0.15 ≥ 0.20, ++++, >0.20.

c Increased total peptide concentration (mg/L): , ≤1.0; +, 1.0 ≥ 4.0; ++, 4.0 ≥ 6.0; +++, 6.0 ≥ 8.0; ++++, >8.0.

d The values corresponding to trypsin are an average of those obtained in all twelve incubations.

### 3.3 Assessment of protein and peptide extraction from A. platensis by the most active peptidases

Table 2 presents the effect of individual peptidases, commercial trypsin, pancreatin, a six-enzyme mixture (MIX6) containing each selected recombinant enzyme, and MIX6 combined with either trypsin (MIX6TRP) or pancreatin (MIX6PAN), on the extraction of protein and peptides from *A. platensis*. No significant differences (*P* > 0.050) were observed between enzymatic treatments and the control for total protein extracted into the algal supernatant. However, enzyme ID 138 demonstrated a significant increase (*P* = 0.036) in total peptides released from microalgal biomass compared to the control. Furthermore, the total protein extracted with enzymes ID 152, 153 and 159 was higher (*P* < 0.050) than that obtained with commercial trypsin and pancreatin. The MIX6, MIX6TRP and MIX6PAN treatments resulted in a significant increase (*P* < 0.001) in total protein extraction yield compared to the use of individual enzymes. The lack of significant differences (*P* > 0.050) between combined and individual peptidases in terms of total protein and peptide concentration suggests that there were no additive or synergistic actions between the enzymes.

**Table 2:** Effect of the most active peptidases, individually or combined, on the concentration of total protein and peptides released into *Arthrospira platensis* supernatant.

Item	Treatments^a^												SEM^b^	*P*-value
	Control	MIX6	MIX6TRP	MIX6PAN	ID113	ID121	ID138	ID152	ID153	ID159	Trypsin	Pancreatin		
Protein (g/L)	0.338^ab^	0.401^ab^	0.381^ab^	0.409^ab^	0.462^ab^	0.447^ab^	0.466^ab^	0.530^a^	0.500^a^	0.548^a^	0.257^b^	0.266^b^	0.0413	0.001
Peptide (mg/L)	44.6^b^	52.6^ab^	53.9^ab^	55.8^ab^	61.7^ab^	60.6^ab^	67.9^a^	54.3^ab^	59.5^ab^	57.3^ab^	60.8^ab^	59.0^ab^	3.70	0.036
Increased protein (g/L)	–	0.063^bcde^	0.043^cde^	0.071^abcd^	0.077^abc^	0.062^bcde^	0.130^abc^	0.194^ab^	0.164^abc^	0.212^a^	−0.081^e^	−0.072^de^	0.0277	<0.001
Increased peptide (mg/L)	–	7.97^abcd^	9.27^abcd^	11.1^abc^	9.04^abcd^	7.89^abcd^	8.09^abcd^	−5.55^d^	−0.26^bcd^	−2.49^cd^	16.2^a^	14.4^ab^	3.06	0.003
Total protein extraction yield (%)	–	1.26^a^	0.85^a^	1.41^a^	−2.83^b^	−2.96^b^	−2.81^b^	−2.29^b^	−2.53^b^	−2.15^b^	−1.60^b^	−1.44^b^	0.434	<0.001

^a,b,c,d,e^ Different superscripts indicate significant differences between peptidases, individually or combined (*P* < 0.05).

a MIX6: ID 113, 121, 138, 152, 153, 159; TRP, trypsin; PAN, pancreatin.

b Standard error of the mean.

### 3.4 Evaluation of hydrolysis and solubility of A. platensis’ proteins for the most active peptidases

**Fig. 1:**
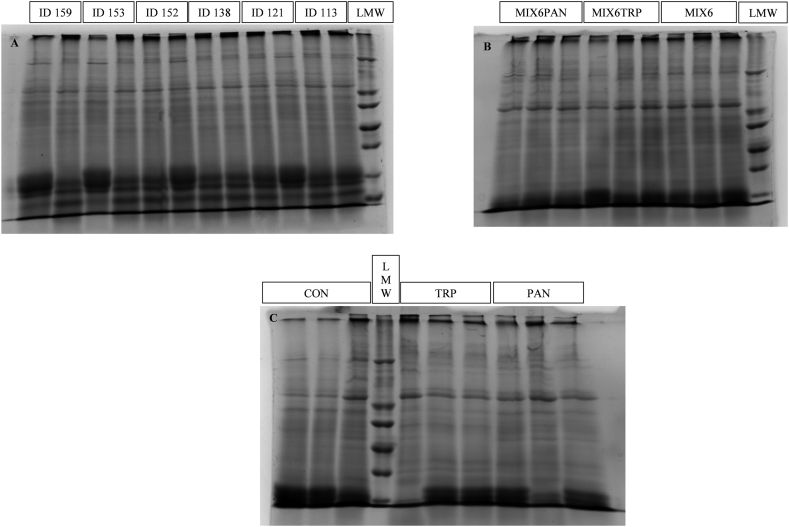
Sodium dodecyl sulphate-polyacrylamide gel electrophoresis using 14 % (w/v) acrylamide gels displaying protein fractions of *Arthrospira platensis* after digestion with individually selected peptidases (cropped) (A); a mixture of six peptidases (MIX6) (ID 113, ID 121, ID 138, ID 152, ID 153 and ID 159), MIX6 combined with trypsin (MIX6TRP) or pancreatin (MIX6PAN) (original Supplementary Fig. 2A) (B); no enzymes (CON, control), trypsin (TRP) or pancreatin (PAN) (original Supplementary Fig. 2B) (C). LMW: low molecular weight protein marker (18–96 kDa) (original Supplementary Fig. 2C).

Table 3 depicts the effect of selected enzymes on total protein and individual protein fractions, as evaluated using SDS-PAGE and observable in gels from Fig. 1A and B, and C (cropped) and in Supplementary Fig. 2A, B and C (uncropped, original). No significant differences were identified (*P* > 0.050) between enzymatic treatments and the control for total soluble protein, a contrast to the results observed for individual protein fractions. Specifically, enzymes ID 152 and ID 153 significantly increased (*P* < 0.001) the quantity of the protein fraction within the range of 18–26 kDa in the algal supernatant, with intermediate values (*P* < 0.050) noted for MIX6, MIX6TRP and MIX6PAN. Conversely, enzyme ID 138, along with trypsin and pancreatin, significantly decreased (*P* < 0.001) the concentration of the protein fraction ranging from 40 to 48 kDa. A similar trend was seen for the proportion of protein fractions but without significant differences (*P* > 0.050) between enzyme mixtures and the control for the first fraction. However, the second fraction showed an increment (*P* < 0.001) with enzymes ID 113, 121, 152, and 159. Additionally, pancreatin only led to a numerical decrease in the percentage of the 40- to 48-kDa fraction compared to the control. Moreover, enzymes ID 152, ID 153 and ID 159 significantly decreased (*P* < 0.001) the proportion of other minor protein fractions.

**Table 3:** Effect of the most active peptidases, individually or combined, on the concentration/proportion of total protein and protein fractions in *Arthrospira platensis* supernatant measured in SDS-PAGE gels.

Item	Treatments^a^												SEM^b^	*P*-value
	Control	MIX6	MIX6TRP	MIX6PAN	ID113	ID121	ID138	ID152	ID153	ID159	Trypsin	Pancreatin		
Protein quantification (g/L)														
Total protein	10.1^abcd^	11.4^a^	10.9^abc^	11.1^ab^	9.15^d^	9.10^d^	9.65^bcd^	9.83^abcd^	9.51^cd^	9.06^d^	9.27^d^	8.85^d^	0.292	<0.001
Protein 18–26 kDa	2.25^cd^	2.48^b^	2.45^b^	2.45^b^	2.09^ef^	2.08^ef^	2.16^de^	2.70^a^	2.69^a^	2.28^c^	2.00^f^	2.02^f^	0.019	<0.001
Protein 40–48 kDa	1.80^a^	1.97^a^	1.95^a^	1.94^a^	1.83^a^	1.86^a^	1.36^b^	1.80^a^	1.76^a^	1.86^a^	1.46^b^	1.47^b^	0.041	<0.001
Other proteins	5.99^abc^	6.91^a^	6.53^ab^	6.72^a^	5.24^bc^	5.17^bc^	6.12^abc^	5.32^bc^	5.06^c^	4.92^c^	5.80^abc^	5.36^bc^	0.259	<0.001
Protein proportion (% total)														
Protein 18–26 kDa	22.5^b^	21.9^b^	22.5^b^	22.0^b^	22.8^b^	22.8^b^	23.6^b^	29.5^a^	29.4^a^	24.9^b^	21.6^b^	22.9^b^	0.623	<0.001
Protein 40–48 kDa	18.0^bc^	17.3^cd^	17.9^bc^	17.5^c^	20.0^a^	20.4^a^	14.9^e^	19.7^a^	19.3^ab^	20.3^a^	15.8^de^	16.6^cde^	0.326	<0.001
Other proteins	59.5^ab^	60.8^ab^	59.7^ab^	60.5^ab^	57.2^bc^	56.8^bc^	61.5^ab^	50.8^d^	51.4^d^	54.8^cd^	62.6^a^	60.5^ab^	0.866	<0.001

^a,b,c,d,e^ Different superscripts indicate significant differences between peptidases, individually or combined (*P* < 0.05).

a MIX6: ID 113, 121, 138, 152, 153, 159; TRP, trypsin; PAN, pancreatin.

b Standard error of the mean.

### 3.5 Assessment of coefficients of protein degradation for A. platensis for the selected peptidases

Table 4 presents the impact of individual and combined peptidases on CPD values, which quantitatively represent the degree of algal protein hydrolysis. Remarkably, MIX6 significantly enhanced (*P* < 0.001) the hydrolysis of total soluble protein compared to enzymes ID 113, 121, 159, trypsin, and pancreatin. Enzymes ID 152 and ID 153 notably increased (*P* < 0.001) the hydrolysis of the protein fraction ranging from 18 to 26 kDa, with intermediate values (*P* < 0.050) observed for MIX6, MIX6TRP and MIX6PAN relative to other treatments. In contrast, enzyme ID 138, trypsin, and pancreatin significantly reduced (*P* < 0.001) CPD values for the protein fraction ranging from 40 to 48 kDa. Additionally, MIX6 demonstrated a numerical increase in the hydrolysis of other minor protein fractions, showing a considerable effect (*P* = 0.002) compared to enzymes ID 153, 159, and pancreatin.

**Table 4:** Effect of the most active peptidases on the coefficients of protein degradation (%) of *Arthrospira platensis* protein fractions.

Item	Treatments^a^											SEM^b^	*P*-value
	MIX6	MIX6TRP	MIX6PAN	ID113	ID121	ID138	ID152	ID153	ID159	Trypsin	Pancreatin		
Total protein	13.4^a^	8.96^abc^	11.0^ab^	−4.58^bcd^	−5.11^cd^	0.52^abcd^	2.44^abcd^	−0.86^abcd^	−5.58^cd^	−7.64^d^	−11.9^d^	3.046	<0.001
Protein 18–26 kDa	10.3^b^	8.88^b^	8.76^b^	−7.66^de^	−8.13^de^	−4.32^cd^	19.4^a^	19.0^a^	0.90^c^	−11.2^e^	−10.4^de^	1.245	<0.001
Protein 40–48 kDa	9.10^a^	8.25^a^	7.93^a^	4.33^a^	6.11^a^	−22.2^b^	3.03^a^	0.68^a^	6.05^a^	−18.9^b^	−18.6^b^	2.454	<0.001
Other proteins	16.2^a^	9.32^abc^	13.1^ab^	−6.13^abc^	−7.41^abc^	9.59^abc^	−4.63^abc^	−9.40^bc^	−11.8^c^	−2.80^abc^	−10.2^c^	4.729	0.002

^a,b,c,d,e^ Different superscripts indicate significant differences between peptidases, individually or combined (*P* < 0.05).

a MIX6: ID 113, 121, 138, 152, 153, 159; TRP, trypsin; PAN, pancreatin.

b Standard error of the mean.

## 4 Discussion

In this study, we carefully selected 192 recombinant peptidases to assess their capacity to extract and hydrolyse proteins from *A. platensis*. To optimize their activities on microalgal compounds, we considered the origin of these enzymes, with 60 derived from freshwater/marine organisms and 72 from hyperthermophilic/thermophilic organisms. While all peptidase families were well represented, the majority of enzymes were focused on unknown substrates. Only 19 peptidases were identified as potentially acting on *A. platensis* cell wall components, including peptidoglycan-associated proteins [2]. These enzymes were produced using a high-throughput platform, allowing 142 peptidases to progress to the initial activity screening stage. While the protein expression rate was substantial at 74 %, the overall efficiency was compromised due to *E. coli* cell wall disruption, which occurred in 26 % (50 out of 192) of all peptidases. Additionally, commercial trypsin was included in the evaluation owing to its broad activity spectrum and demonstrated substantial peptide production (10.6 mg/L) when applied to *A. platensis* biomass. Ultimately, we identified 6 recombinant peptidases and trypsin as the most active enzymes for *A. platensis* protein hydrolysis. These enzymes were combined to maximize activity and mixed with commercial porcine pancreatin, which had shown effective hydrolysis of *A. platensis* proteins (average of 81 % of crude protein digestibility) when paired with pepsin in previous *in vitro* digestibility assays [12].

Our findings revealed that recombinant peptidases did not significantly affect the total protein extraction from *A. platensis* biomass. However, enzymes ID 152, ID 153 and ID 159 yielded the highest extraction values, whereas trypsin and pancreatin resulted in the lowest. It has been previously reported that microalga proteins, specifically *c*-phycocyanin (which constitutes up to 20 % of dried biomass), exhibit lower sensitivity to trypsin compared to other enzymes, such as pepsin [15]. Interestingly, enzyme ID 138 substantially increased the release of total peptides into the algal supernatant compared to the control, albeit without significant variations compared to other enzymatic treatments. While the mixtures MIX6, MIX6TRP and MIX6PAN elevated total protein extraction yield relative to other treatments, the lack of significant differences between these mixtures and the individual peptidases regarding the release of total protein and peptides from *A. platensis* biomass suggests an absence of synergistic or additive effects between the enzymes.

Enzyme ID 138 (UniProt: D7B6J8), produced by *Nocardiopsis dassonvillei*, shows homology with the Htra2/pepD peptidase from *Mycobacterium tuberculosis* (MEROPS accession number: MER0004673). This enzyme is a serine chymotrypsin-like endopeptidase belonging to the S1 family. The characteristic catalytic triad residues—His, Asp, and Ser—are present in all members of subclan PA(S). This enzyme cleaves the substrate at the P1 position, following a hydrophobic amino acid. The specific activity of this enzyme in the hydrolysis of *A. platensis* proteins and the subsequent production of peptides remains unknown. Nevertheless, Verdasco-Martín, Díaz-Lozano [34] and Otero and Verdasco-Martín [16] have reported significant extraction of short peptides (ranging from 0.438 to 1.493 kDa) and amino acids from *A. platensis* using another serine endopeptidase, i.e. alcalase. Similarly, Villaró, Jiménez-Márquez [17] confirmed the effectiveness of alcalase in hydrolysing *A. platensis* proteins, showing a higher degree of hydrolysis compared to pepsin, but with activity comparable to cysteine peptidases such as papain and ficin.

When evaluating the protein fraction profile via SDS-PAGE gel, we found that enzymes ID 152 and ID 153 amplified the concentration and proportion of the protein fraction between 18 and 26 kDa, likely corresponding to *α*- and *β*-subunits of allophycocyanin and *c*-phycocyanin [5]. Consequently, the CPD values for this protein fraction were higher with these peptidases and intermediate with MIX6, compared to other treatments. This result suggests that enzymes ID 152 and 153 may facilitate the hydrolysis of phycocyanin, with the enzyme mixture leading to some degradation. Enzyme ID 152 (Genbank: MBE0363726.1), a hypothetical protein produced by *Pseudoalteromonas ulvae* UL12, was originally isolated from the marine macroalga, *Ulva lactuca* [35]. *Pseudoalteromonas* species have been identified as ulvan-degrading bacteria [36]. Ulvan, a branched polysaccharide in *U. lactuca* cell walls, consists of disaccharide repeating units, including ulvanobiuronic acids and ulvanobioses [37]. These bacteria can produce polysaccharide lyases from family 25, capable of depolymerizing ulvan into di- and tetrasaccharides of uronic acids, as reported by Ulaganathan, Boniecki [38] and suggested by Costa, Pio [25]. Consequently, it is plausible that enzyme ID 152 may hydrolyse proteins, such as glycoproteins, associated with cell wall polysaccharides composed of monosaccharides and uronic acids in *A. platensis* [4]. This is corroborated by Otero and Verdasco-Martín [16], who observed the release of phycocyanin from microalga biomass using a commercial carbohydrase with exo-1,3-glucanase activity. Earlier, Van Eykelenburg, Fuchs [3] speculated about the presence of peptide bridges between the carbohydrate chains of peptidoglycans, suggesting a linkage between peptide residues and cell wall polysaccharides in *A. platensis*. Additionally, enzyme ID 153 is a cellulosomal serine endopeptidase (cprA) (Genbank: AM231039.1; UniProt: Q2HPT9) produced by the thermophilic bacterium *Acetivibrio thermocellus*. This peptidase, belonging to the subtilase (S8A) subfamily, shares homology with an enzyme from *Pseudoalteromonas* sp.: deseasin MCP-01. This latter enzyme contains a catalytic triad—"Asp, His, and Ser” residues—and can hydrolyse various substrates such as casein, bovine serum albumin, and gelatine [39]. However, its activity on microalga proteins remains unknown. Given that enzyme ID 153 is a serine endopeptidase, akin to enzyme ID 138, further exploration of its activity on *A. platensis* proteins is warranted.

## 5 Conclusion and future perspectives

The present investigation has provided valuable insights into the hydrolysis of proteins from *A. platensis* using specific recombinant enzymes. Among the six-enzyme mixture tested, enzyme ID 138, a serine-peptidase, demonstrated significant potential by enhancing the release of total peptides into *A. platensis* supernatant. Additionally, enzymes ID 152, derived from a marine bacterium, and ID 153, a serine-peptidase, showed remarkable efficacy in amplifying the extraction and hydrolysis of the protein fraction within the range of 18–26 kDa, likely corresponding to a phycocyanin fraction. These observations underscore the potential of serine-peptidases in effectively hydrolysing *A. platensis* proteins, particularly protein-pigment complexes, and liberating peptides.

The results of this study lay the groundwork for future investigations, which should encompass a comprehensive characterization of the peptidases present in MIX6, with a particular focus on enzymes ID 138, ID 152, and ID 153. Further studies should evaluate the practical effectiveness of these enzymes in enhancing the bioaccessibility of microalgal proteins when integrated as supplements in monogastric animal diets that incorporate *A. platensis*. This research offers a pathway to optimize the nutritional value of such diets, thereby contributing to more effective utilization of this valuable microalgal protein source.

## CRediT authorship contribution statement

**Maria P. Spínola:** Methodology, Formal analysis, Data curation. **Mónica M. Costa:** Writing – original draft, Methodology, Formal analysis, Data curation. **Rita S. Simões:** Methodology, Formal analysis, Data curation. **Vânia O. Fernandes:** Data curation. **Vânia Cardoso:** Data curation. **Virgínia M.R. Pires:** Data curation. **Cláudia Afonso:** Data curation. **Carlos Cardoso:** Data curation. **Narcisa M. Bandarra:** Data curation. **Carlos M.G.A. Fontes:** Methodology, Formal analysis, Data curation. **José A.M. Prates:** Writing – review & editing, Supervision, Project administration, Funding acquisition, Conceptualization.

## Additional information

No additional information is available for this paper.

## Data availability statement

All data generated or analysed during this study are included in this published article.

## Declaration of competing interest

The authors declare that they have no known competing financial interests or personal relationships that could have appeared to influence the work reported in this paper.

## References

[1] Spínola M.P., Costa M.M., Prates J.A.M. (2022). Digestive constraints of Arthrospira platensis in poultry and swine feeding. Foods.

[2] Van Eykelenburg C. (1977). On the morphology and ultrastructure of the cell wall of Spirulina platensis. Antonie Leeuwenhoek.

[3] Van Eykelenburg C., Fuchs A., Schmidt G.H. (1980). Some theoretical considerations on the in vitro shape of the cross-walls in Spirulina spp. J. Theor. Biol..

[4] Bernaerts T.M.M. (2018). Comparison of microalgal biomasses as functional food ingredients: focus on the composition of cell wall related polysaccharides. Algal Res..

[5] Böcker L. (2020). Time-temperature-resolved functional and structural changes of phycocyanin extracted from Arthrospira platensis/Spirulina. Food Chem..

[6] Buecker S. (2022). Thermal and acidic denaturation of phycocyanin from Arthrospira platensis: effects of complexation with λ-carrageenan on blue color stability. Food Chem..

[7] Safi C. (2014). Aqueous extraction of proteins from microalgae: effect of different cell disruption methods. Algal Res..

[8] Lupatini A.L. (2017). Protein and carbohydrate extraction from S. platensis biomass by ultrasound and mechanical agitation. Food Res. Int..

[9] Tavanandi H.A., Chandralekha Devi A., Raghavarao K. (2018). A newer approach for the primary extraction of allophycocyanin with high purity and yield from dry biomass of Arthrospira platensis. Sep. Purif. Technol..

[10] Tavanandi H.A., Vanjari P., Raghavarao K.S.M.S. (2019). Synergistic method for extraction of high purity allophycocyanin from dry biomass of Arthrospira platensis and utilization of spent biomass for recovery of carotenoids. Sep. Purif. Technol..

[11] Coelho D. (2020). A two-enzyme constituted mixture to improve the degradation of Arthrospira platensis microalga cell wall for monogastric diets. J. Anim. Physiol. Anim. Nutr..

[12] Niccolai A. (2019). Microalgae of interest as food source: biochemical composition and digestibility. Algal Res..

[13] Evans A.M., Smith D.L., Moritz J.S. (2015). Effects of algae incorporation into broiler starter diet formulations on nutrient digestibility and 3 to 21 d bird performance. J. Appl. Poultry Res..

[14] Pestana J.M. (2020). Impact of dietary incorporation of Spirulina (Arthrospira platensis) and exogenous enzymes on broiler performance, carcass traits, and meat quality. Poultry Sci..

[15] Aiello G. (2019). Chemical and biological characterization of Spirulina protein hydrolysates: focus on ACE and DPP-IV activities modulation. J. Funct.Foods.

[16] Otero C., Verdasco-Martín C.M. (2023). Preparation and characterization of a multicomponent Arthrospira platensis biomass hydrolysate with superior anti-hypertensive, anti-hyperlipidemic and antioxidant activities via selective proteolysis. Mar. Drugs.

[17] Villaró S. (2023). Production of enzymatic hydrolysates with in vitro antioxidant, antihypertensive, and antidiabetic properties from proteins derived from Arthrospira platensis. Food Res. Int..

[18] Dinda B. (2015). Oroxylum indicum (L.) Kurz, an important Asian traditional medicine: from traditional uses to scientific data for its commercial exploitation. J. Ethnopharmacol..

[19] Xiang Z. (2023). Antioxidant peptides from edible aquatic animals: preparation method, mechanism of action, and structure-activity relationships. Food Chem..

[20] Shi Y. (2018). The genus Ficus (Moraceae) used in diet: its plant diversity, distribution, traditional uses and ethnopharmacological importance. J. Ethnopharmacol..

[21] Lafarga T., Hayes M. (2014). Bioactive peptides from meat muscle and by-products: generation, functionality and application as functional ingredients. Meat Sci..

[22] Coelho D. (2019). Novel combination of feed enzymes to improve the degradation of Chlorella vulgaris recalcitrant cell wall. Sci. Rep..

[23] Saez N.J. (2014). High throughput quantitative expression screening and purification applied to recombinant disulfide-rich venom proteins produced in E. coli. J. Vis. Exp..

[24] Costa M. (2021). An individual alginate lyase is effective in the disruption of Laminaria digitata recalcitrant cell wall. Sci. Rep..

[25] Costa M.M. (2022). Recalcitrant cell wall of Ulva lactuca seaweed is degraded by a single ulvan lyase from family 25 of polysaccharide lyases. Anim. Nutr..

[26] Costa M.M., Spínola M.P., Prates J.A.M. (2023). Combination of mechanical/physical pretreatments with trypsin or pancreatin on Arthrospira platensis protein degradation. Agriculture.

[27] Bradford M.M. (1976). A rapid and sensitive method for the quantitation of microgram quantities of protein utilizing the principle of protein-dye binding. Anal. Biochem..

[28] Spínola M.P., Costa M.M., Prates J.A.M. (2023). Studies on the impact of selected pretreatments on protein solubility of Arthrospira platensis microalga. Agriculture.

[29] Postma P.R. (2015). Mild disintegration of the green microalgae Chlorella vulgaris using bead milling. Bioresour. Technol..

[30] Sedighi M. (2019). Enzymatic hydrolysis of microalgae proteins using serine proteases: a study to characterize kinetic parameters. Food Chem..

[31] Vizcaíno A.J. (2019). Differential hydrolysis of proteins of four microalgae by the digestive enzymes of gilthead sea bream and Senegalese sole. Algal Res..

[32] Alarcón F.J., Moyano F.J., Díaz M. (2001). Use of SDS-PAGE in the assessment of protein hydrolysis by fish digestive enzymes. Aquacult. Int..

[33] Weber S. (2022). Insights into cell wall disintegration of Chlorella vulgaris. PLoS One.

[34] Verdasco-Martín C.M., Díaz-Lozano A., Otero C. (2020). Advantageous enzyme selective extraction process of essential Spirulina oil. Catal. Today.

[35] Egan S., Holmström C., Kjelleberg S. (2001). Pseudoalteromonas ulvae sp. nov., a bacterium with antifouling activities isolated from the surface of a marine alga. Int. J. Syst. Evol. Microbiol..

[36] Kopel M. (2014). Draft genome sequence of Pseudoalteromonas sp. strain PLSV, an ulvan-degrading bacterium. Genome Announc..

[37] Lahaye M., Robic A. (2007). Structure and functional properties of ulvan, a polysaccharide from green seaweeds. Biomacromolecules.

[38] Ulaganathan T. (2017). New ulvan-degrading polysaccharide lyase family: structure and catalytic mechanism suggests convergent evolution of active site architecture. ACS Chem. Biol..

[39] Chen X.L. (2007). A novel type of subtilase from the psychrotolerant bacterium Pseudoalteromonas sp. SM9913: catalytic and structural properties of deseasin MCP-01. Microbiology.

