# Supplementary material for: Improving protein hydrolysis and digestibility in Arthrospira platensis biomass through recombinant peptidases (EC 3.4): Opportunities for monogastric animal diets
Source: Heliyon. 2024 Dec 25;11(1):e41460. doi: 10.1016/j.heliyon.2024.e41460 (PMC11742843; doi:10.1016/j.heliyon.2024.e41460)
Supplement: Multimedia component 1 [file mmc1.docx]

Supplementary Material

**Improving protein hydrolysis and digestibility in Arthrospira platensis biomass through recombinant peptidases (EC 3.4): opportunities for monogastric animal diets**

Maria P. Spínola^1,2^+, Mónica M. Costa^1,2^+, Rita S. Simões^1,2,3^+, Vânia O. Fernandes^1,2,3^, Vânia Cardoso^1,2,3^, Virgínia M. R. Pires^1,2,3^, Cláudia Afonso^4,5^, Carlos Cardoso^4,5^, Narcisa M. Bandarra^4,5^, Carlos M. G. A. Fontes^1,2,3^, José A. M. Prates^1,2*^

*** Correspondence:** José António Mestre Prates: [japrates@fmv.ulisboa.pt](mailto:japrates@fmv.ulisboa.pt)

**+** These authors contributed equally to this work and share first authorship

# Supplementary Figures


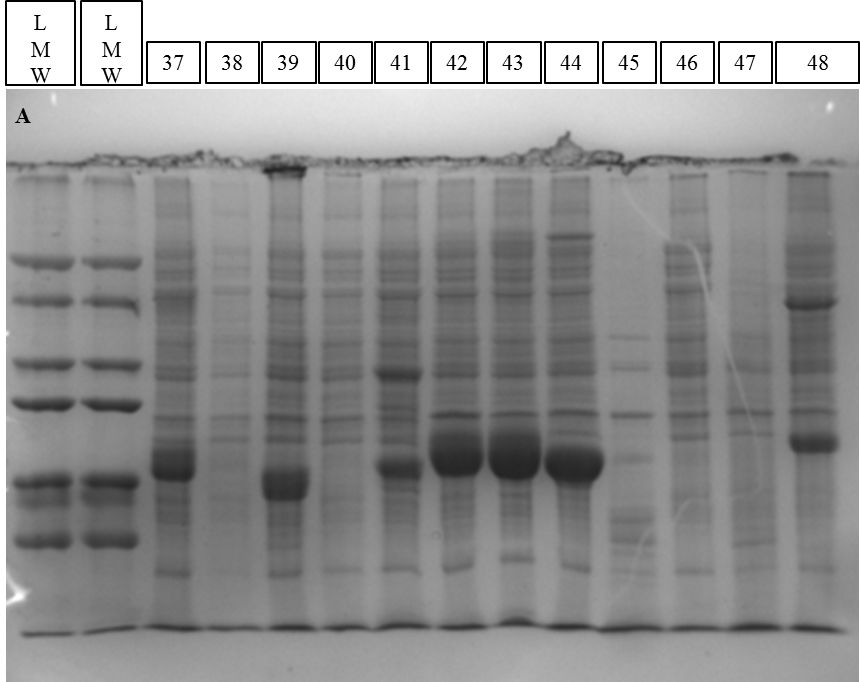


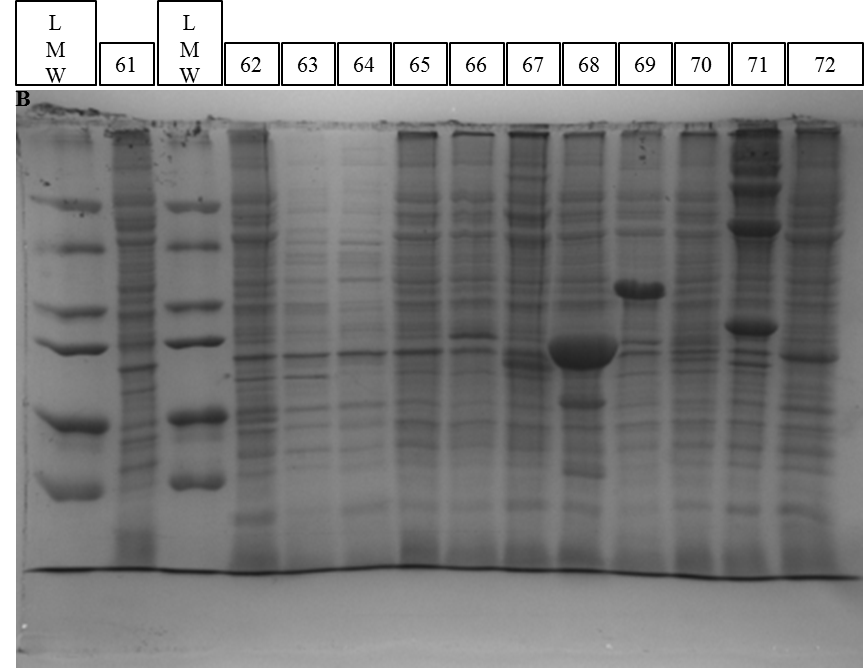


**Supplementary Figure 1.** Sodium dodecyl sulphate-polyacrylamide gel electrophoresis (SDS-PAGE) in 12% (w/v) acrylamide gels displaying the fragment bands obtained by electrophoresis of soluble fractions of recombinant proteins (the numbers correspond to enzyme identification number, ID). Protein fractions of enzymes with ID 37 to 48 (**A**) and ID 61 to 72 (**B**), LMW: Low Molecular Weight protein marker.


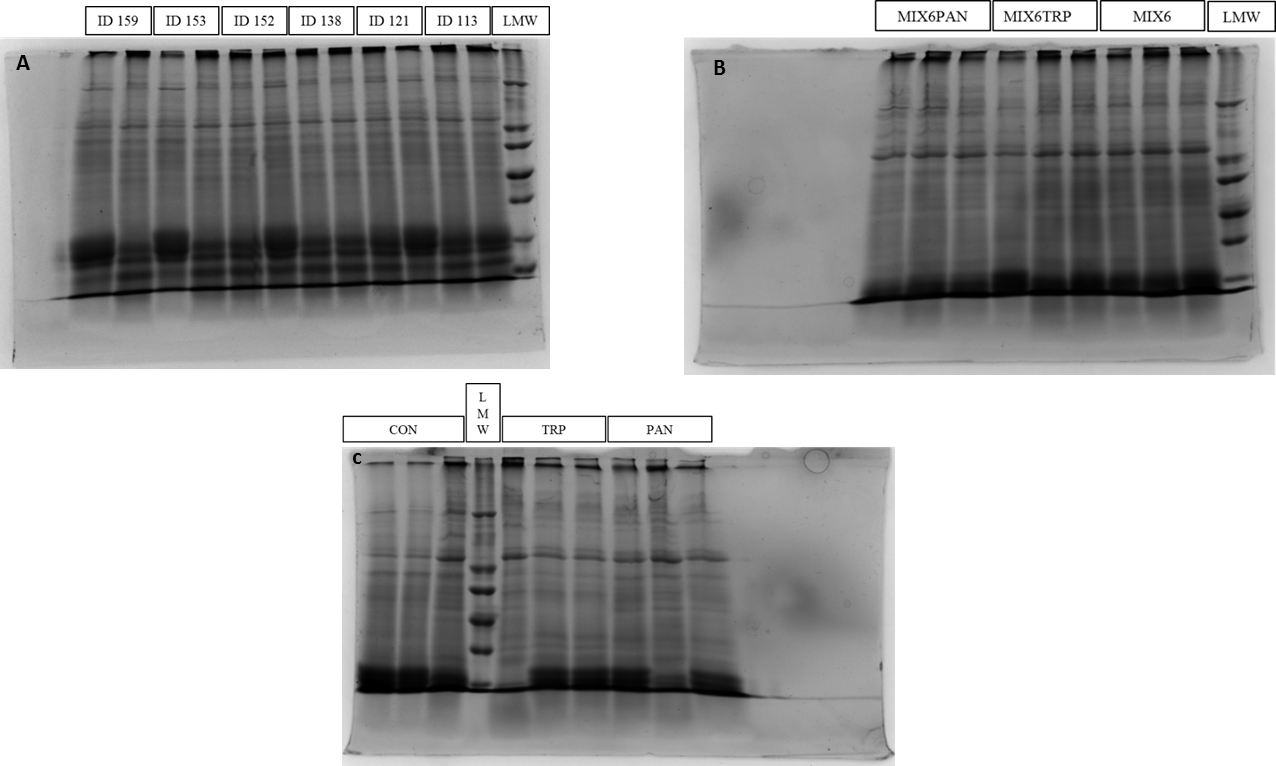


**Supplementary Figure 2.**  Sodium dodecyl sulphate-polyacrylamide gel electrophoresis using 14% (w/v) acrylamide gels displaying protein fractions of *Arthrospira platensis* after digestion with individually selected peptidases (uncropped gels) (**A**); a mixture of six peptidases (MIX6) (ID 113, ID 121, ID 138, ID 152, ID 153 and ID 159), MIX6 combined with trypsin (MIX6TRP) or pancreatin (MIX6PAN) (**B**); no enzymes (CON, control), trypsin (TRP) or pancreatin (PAN) (**C**). LMW: low molecular weight protein marker (18 to 96 kDa). This are the original figure for Figure 1, within the manuscript (that is cropped).
